# Supplementary material for: Ramadan during pregnancy and neonatal health—Fasting, dietary composition and sleep patterns
Source: PLoS One. 2023 Feb 15;18(2):e0281051. doi: 10.1371/journal.pone.0281051 (PMC9931121; doi:10.1371/journal.pone.0281051)
Supplement: S2 File — (DOCX) [file pone.0281051.s002.docx]

## Online Appendix II – Background: Oster Method

The main concern when comparing birth outcomes of fasting vs non-fasting pregnant Muslims is that fasting is not randomly assigned. Consequently, our set of covariates may not take out all relevant differences between the fasting and the non-fasting group. In such situations, the Oster test statistic can be calculated to test how likely it is that results are driven by residual confounding ([Oster 2017](#_ENREF_36)). The Oster test statistic compares the raw fasting-birthweight association with the adjusted association from the regression analysis. Taking into account how the coefficient of interest, as well as explained variance (R²) change, the test statistic indicates how important residual confounders would have to be in order to explain away the reported effect to the point where the null hypothesis (no effect of maternal fasting on birthweight) would no longer be rejected. For example, a test statistic value of 3 would indicate that residual confounders would need to be three times as important as the covariates included in the model to explain away the fasting-birthweight association. The Oster method requires to set the maximum $R^{2}$ that is theoretically reachable in a model that would include all possible (observable and unobservable) determinants of the dependent variable. We follow Oster (2017) in setting this $R_{max}$ as $1ˑ3*\tilde{R}$, with $\tilde{R}$ being the $R^{2}$ from our adjusted regression, but as a sensitivity analysis, we also use $R_{max}=1$.

Compared to the raw association (-66ˑ67g, 95% CI: -193ˑ28; 59ˑ97, cf. Table 2), adding the covariates to the regression model leads to a stronger association between fasting and birthweight. The Oster test statistic shows that residual confounders would not only have to be over eighteen times as important as the included covariates in explaining the outcome variable, but they would have to work in the opposite direction from the included covariates in order to eliminate the detected effects on birthweight ($\delta=-18ˑ68$). In the robustness test using the more conservative $R_{max}=1$ instead of $R_{max}=1ˑ3*0ˑ41=0ˑ53$ (with 0.41 being the R² from our regression), this test statistic changes to $\delta=-4ˑ17$, which would still require residual confounders to be more than four times as important as the included covariates, and to work into the opposite direction. It is thus very unlikely that the observed fasting-birthweight association is due to residual confounding.
