# Supplementary material for: Ramadan during pregnancy and neonatal health—Fasting, dietary composition and sleep patterns
Source: PLoS One. 2023 Feb 15;18(2):e0281051. doi: 10.1371/journal.pone.0281051 (PMC9931121; doi:10.1371/journal.pone.0281051)
Supplement: S1 Fig — This figure shows the results of three adjusted regressions. The reference group are non-fasting women. Gestational age at birth is measured in completed weeks of gestation. (DOCX) [file pone.0281051.s003.docx]

Supporting Figure 1. Fasting and Gestational Age at Birth (in Weeks)


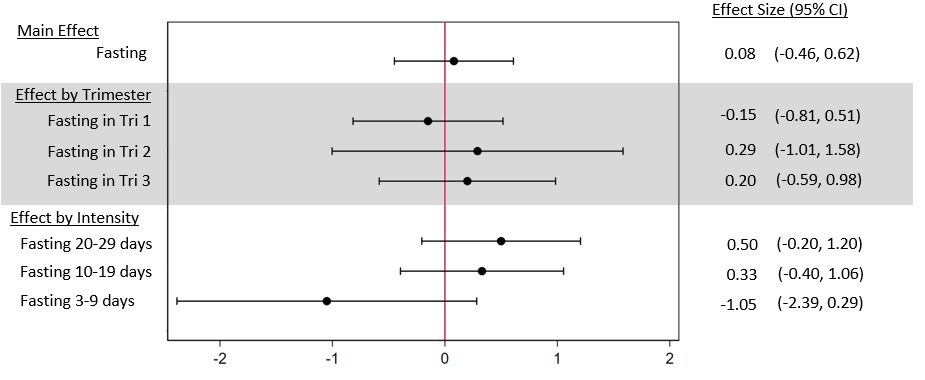


This figure shows the results of three adjusted regressions. The reference group are non-fasting women. Gestational age at birth is measured in completed weeks of gestation.
