# Supplementary material for: Ramadan during pregnancy and neonatal health—Fasting, dietary composition and sleep patterns
Source: PLoS One. 2023 Feb 15;18(2):e0281051. doi: 10.1371/journal.pone.0281051 (PMC9931121; doi:10.1371/journal.pone.0281051)
Supplement: S2 Fig — This figure shows the results of two adjusted regressions. The respective reference groups are indicated in the figure. Gestational age is measured in completed weeks of gestation. (DOCX) [file pone.0281051.s004.docx]

Supporting Figure 2. Fasting, Sleep and Dietary Adaptations and Gestational Age at Birth in Weeks


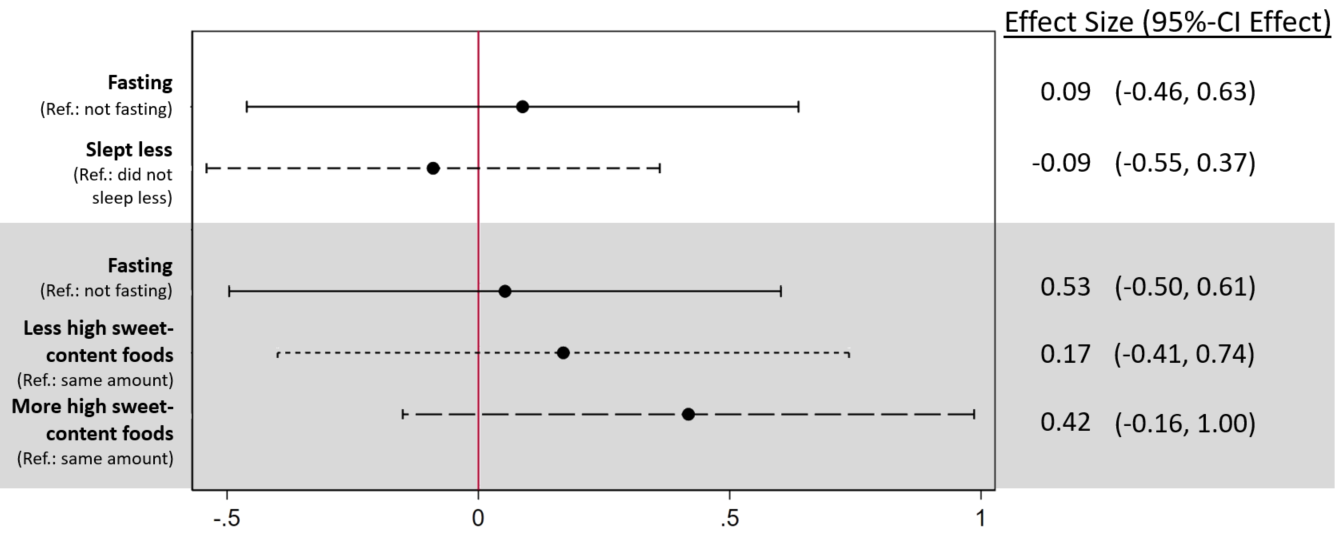


This figure shows the results of two adjusted regressions. The respective reference groups are indicated in the figure. Gestational age is measured in completed weeks of gestation.
