# Supplementary material for: Ramadan during pregnancy and neonatal health—Fasting, dietary composition and sleep patterns
Source: PLoS One. 2023 Feb 15;18(2):e0281051. doi: 10.1371/journal.pone.0281051 (PMC9931121; doi:10.1371/journal.pone.0281051)
Supplement: S3 Fig — This figure shows the results of four adjusted regressions. In each regression, the fasting variable is interacted with the depicted variable. The reference group is always the offspring of mothers who did not fast. Gestational age at birth is measured in completed weeks. (DOCX) [file pone.0281051.s005.docx]

Supporting Figure 3. Effect of Fasting on Gestational Age at Birth (in Weeks) Interacted with Dietary Intake and Sleep during Ramadan


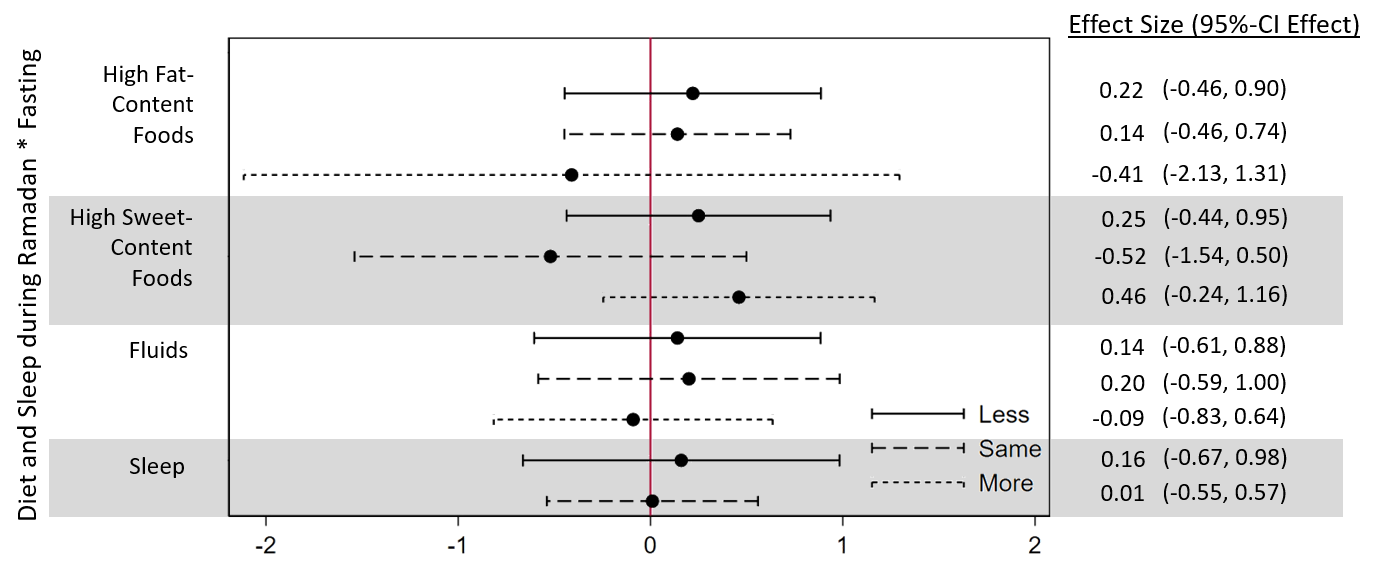


This figure shows the results of four adjusted regressions. In each regression, the fasting variable is interacted with the depicted variable. The reference group is always the offspring of mothers who did not fast. Gestational age at birth is measured in completed weeks.
