# Supplementary material for: Ramadan during pregnancy and neonatal health—Fasting, dietary composition and sleep patterns
Source: PLoS One. 2023 Feb 15;18(2):e0281051. doi: 10.1371/journal.pone.0281051 (PMC9931121; doi:10.1371/journal.pone.0281051)
Supplement: S1 Table — Note: Each column shows the results from a separate regression. Each regression includes interaction terms of the fasting by trimester indicators with changes in dietary intake (columns 1–3) and sleep patterns (column 4). Note that for sleep patterns, the comparison is only less sleep vs. unchanged sleep patterns, whereas changes in dietary intake were subdivided into the answer categories less/same/more. Robust standard errors are reported in parentheses. Significance levels: ***p<0.01, **p<0.05, *p<0.1. (DOCX) [file pone.0281051.s006.docx]

Supporting Table 1. Regressions of Birthweight on Fasting Interacted with Nutritional Intake and Sleep during Ramadan, by Pregnancy Trimester of Overlap with Ramadan

|  | Interaction of Fasting with Dietary Intake / Sleep Patterns | | | |
| --- | --- | --- | --- | --- |
|  | (1) | (2) | (3) | (4) |
|  | **High Fat-Content Foods** | **High Sweet-Content Foods** | **Fluids** | **Sleep** |
| **Birthweight** (in grams) |  |  |  |  |
| **Fasted in Trimester 1** |  |  |  |  |
| Less*Trimester 1 | -343.60*** | -386.14*** | -265.45** | -392.86*** |
|  | (115.46) | (121.23) | (119.43) | (107.56) |
| Same*Trimester 1 | -422.22*** | -356.43*** | -344.06** | -302.94*** |
|  | (102.98) | (125.24) | (114.46) | (101.92) |
| More*Trimester 1 | -59.80 | -239.12** | -379.38*** |  |
|  | (163.80) | (108.71) | (123.48) |  |
| **Fasted in Trimester 2** |  |  |  |  |
| Less*Trimester 2 | -131.46 | -109.80 | -106.96 | 14.91 |
|  | (161.67) | (201.57) | (177.03) | (179.32) |
| Same*Trimester 2 | -16.21 | 124.73 | 71.24 | -21.41 |
|  | (151.29) | (162.75) | (192.34) | (119.02) |
| More*Trimester 2 | 197.24 | -95.83 | -61.42 |  |
|  | (280.86) | (185.96) | (129.79) |  |
| **Fasted in Trimester 3** |  |  |  |  |
| Less*Trimester 3 | -34.10 | 32.56 | -223.39* | -205.18** |
|  | (153.61) | (135.40) | (120.84) | (93.15) |
| Same*Trimester 3 | -7.57 | 27.40 | 20.86 | 206.40* |
|  | (116.15) | (157.99) | (144.58) | (117.57) |
| More*Trimester 3 | 326.44* | 96.26 | 132.00 |  |
|  | (194.91) | (165.94) | (129.70) |  |
| Note: Each column shows the results from a separate regression. Each regression includes interaction terms of the fasting by trimester indicators with changes in dietary intake (columns 1-3) and sleep patterns (column 4). Note that for sleep patterns, the comparison is only less sleep vs. unchanged sleep patterns, whereas changes in dietary intake were subdivided into the answer categories less/same/more. Robust standard errors are reported in parentheses.  Significance levels: ***p<0.01, **p<0.05, *p<0.1. | | | | |
